# Remote physiological signal recovery with efficient spatio-temporal modeling

**DOI:** 10.3389/fphys.2024.1428351

**Published:** 2024-10-14

**Authors:** Bochao Zou, Yu Zhao, Xiaocheng Hu, Changyu He, Tianwa Yang

**Affiliations:** ^1^ School of Computer and Communication Engineering, University of Science and Technology Beijing, Beijing, China; ^2^ Shunde Graduate School of University of Science and Technology Beijing, Beijing, Guangdong, China; ^3^ Key Laboratory of Complex System Control Theory and Application, Tianjin University of Technology, Tianjin, China; ^4^ China Academy of Electronics and Information Technology, Beijing, China; ^5^ China University of Political Science and Law, Beijing, China

**Keywords:** remote photoplethysmography, physiological measurements, central difference convolution, multi-task, contactless

## Abstract

Contactless physiological signal measurement has great applications in various fields, such as affective computing and health monitoring. Physiological measurements based on remote photoplethysmography (rPPG) are realized by capturing the weak periodic color changes. The changes are caused by the variation in the light absorption of skin surface during systole and diastole stages of a functioning heart. This measurement mode has advantages of contactless measurement, simple operation, low cost, etc. In recent years, several deep learning-based rPPG measurement methods have been proposed. However, the features learned by deep learning models are vulnerable to motion and illumination artefacts, and are unable to fully exploit the intrinsic temporal characteristics of the rPPG. This paper presents an efficient spatiotemporal modeling-based rPPG recovery method for physiological signal measurements. First, two modules are utilized in the rPPG task: 1) 3D central difference convolution for temporal context modeling with enhanced representation and generalization capacity, and 2) Huber loss for robust intensity-level rPPG recovery. Second, a dual branch structure for both motion and appearance modeling and a soft attention mask are adapted to take full advantage of the central difference convolution. Third, a multi-task setting for joint cardiac and respiratory signals measurements is introduced to benefit from the internal relevance between two physiological signals. Last, extensive experiments performed on three public databases show that the proposed method outperforms prior state-of-the-art methods with the Pearson’s correlation coefficient higher than 0.96 on all three datasets. The generalization ability of the proposed method is also evaluated by cross-database and video compression experiments. The effectiveness and necessity of each module are confirmed by ablation studies.

## 1 Introduction

The continuous development of modern society is constantly improving the living standards of people. However, in the meantime, the incidence rate of cardiovascular disease is increasing, which may be caused by the increased work pressure and faster pace of life. The detection of human physiological indicators is of great importance for sensing both affect and health status (Loh et al., 2022; Gupta et al., 2022). However, traditional physiological measurement methods are mostly contact-based, which have the following shortcomings: They are not applicable in some specific application scenarios, such as analyzing the cognitive pressure experienced by interpreters during simultaneous translation, monitoring the psychological states of criminal suspects during interrogation, etc. Moreover, contact measurement requires active cooperation of the tested person. When there is a deviation in the position of the measuring instrument in contact with the skin, it can easily cause a large deviation in the measurement results (Zaunseder et al., 2017; Kumar et al., 2023). In addition, although the electrocardiograph provides accurate measurements, it is relatively expensive and requires to be operated by professionals, which is not suitable for daily physiological measurements.

Plethysmography is the detection of the cardio-vascular pulse wave accomplished by methods such as variations in air pressure or impedance (Verkruysse et al., 2008). Remote photo-plethysmography (PPG) uses light reflectance. This technology can provide contactless monitoring of human cardiac activities by capturing the pulse-induced periodic weak color variations on the skin surface through a conventional camera (McDuff, 2022; Wang et al., 2017; Gupta et al., 2020; Ryu et al., 2021). It is based on the principle that the blood absorbs more light than the surrounding tissues and, therefore, changes in blood volume (caused by systole and diastole stages of the heart) affect transmission and reflectance (Song et al., 2020). Although this pulse-induced variation is subtle, it can be remotely measured on the human face with normal ambient light and a consumer-level camera from a distance of several meters (Verkruysse et al., 2008). The rPPG has many advantages, such as contactless measurement, simple operation, low cost, etc. It provides a new solution for physiological signal measurement and its applications in affective computing (Dasari et al., 2021).

Thanks to the prospective development of computer vision techniques, the subtle change in skin appearance caused by cardiac activities can be detected by low-cost cameras (Verkruysse et al., 2008). Classic signal processing has proved the feasibility of rPPG-based heart rate measurement with the initial success of prototype methods (Yue et al., 2020). However, these methods often show degradation in the presence of artifacts, such as movements, lighting variations, and different skin tones (Shao et al., 2021). With the extensive applications of deep learning in various research fields, such as biometrics (Labati et al., 2022), affective computing (Jung and Sejnowski, 2022), and internet-of-things (Yao et al., 2018), recent studies have also begun to focus on deep learning-based rPPG due to its better representation ability (McDuff, 2022). Several deep learning models, such as convolutional neural network (both 2D and 3D (Zhan et al., 2020)) and recurrent neural network (gated recurrent unit (GRU) (Niu et al., 2019a) and long short-term memory (LSTM) (Hill et al., 2021)), have been successfully applied in the rPPG recovery tasks. However, deep learning-based rPPG can still not effectively model the spatio-temporal information (Ren et al., 2021).

A method of modeling spatio-temporal information by generating a feature map, called STMap (Niu et al., 2019b; Niu et al., 2020; Lu et al., 2021), requires preprocessing including face detection, facial landmarks localization, face alignment, skin segmentation, and color space transformation, which are considerably complicated. Besides, some existing methods directly regress a value, such as heart rate, as the final output instead of recovering the whole waveform, whereas the waveform could be helpful for further analysis of more refined physiological indicators. Furthermore, various loss functions such as L1 loss (mean absolute error, MAE) (Niu et al., 2019a), L2 loss (root mean of squared errors, RMSE) (Tsou et al., 2020), negative Pearson correlation coefficient (Yu et al., 2020a), or more complicated losses with different combinations of these losses exist (Lu et al., 2021). However, the comparison of different loss functions is rarely studied.

Motivated by the above discussion, this paper aims to realize robust contactless physiological signal measurements. Thereby, we propose an rPPG waveform recovery method based on efficient spatiotemporal modeling. It is achieved through a three-dimensional central difference convolution (3D-CDC) operator (Yu et al., 2021) with a dual branch structure composed of motion and appearance branches, as well as a soft attention mask that assigns higher weights to the skin regions with stronger physiological signals. The 3D-CDC can effectively describe intrinsic patterns through the combination of gradient and intensity information. Moreover, to the best of our knowledge, we introduce Huber loss (Wang et al., 2022) for the first time in the rPPG task, which combines the advantage of both L1 and L2 losses, and shows better performance than using these losses separately.

This paper is an extended version of our conference paper (Zhao et al., 2021). The following are the main differences with respect to (Zhao et al., 2021): 1) We propose a multi-task 3D-CDC for pulse wave and respiration wave joint measurement in addition to heart rate measurement; 2) Ablation studies are performed to show the effectiveness of each module, e.g., 3D-CDC, dual branches architecture, and soft attention mask; 3) Robustness of the proposed method with respect to video compression is evaluated and compared with other methods; 4) Extended quantitative and qualitative analyses are provided. The current paper includes additional experiments, data, and interpretation, which have added value to the work proposed in (Yu et al., 2020a).

Our main contributions are as follows:

• An accurate rPPG measurement method based on a 3D-CDC attention network for efficient spatio-temporal modeling is proposed. The utilized 3D-CDC operator can extract temporal context by aggregating temporal difference information.
• Huber loss is adapted as the loss function for rPPG measurements. By evaluating different loss functions and their combinations, we show that better performance is achieved with Huber loss alone by focusing on the intensity level constraint.
• A multi-task variant of the proposed method for joint measurement of cardiac and respiratory activities is developed. It has the advantage of sharing information between related physiological signals, which can further improve accuracy while reducing computational costs.
• Extensive experiments show superior performance on public databases. Both cross-database evaluation and ablation studies are conducted, as well as the effects of video compression are evaluated, which proves the effectiveness and robustness of the proposed method.

The rest of the paper is organized as follows: Section 2 provides the related work and Section 3 gives details about the framework and each module. Section 4 introduces the evaluation settings and implementation details. Section 5 provides the performance of the proposed models on public databases and rigorous ablation studies. Finally, the paper is concluded in Section 6.

## 2 Related work

### 2.1 Signal separation-based rPPG

The remote physiological signal detection method based on rPPG is favored by researchers because it is non-invasive and can obtain physiological signals without any direct contact with the subject’s skin. The underlying mechanism is the delivery of blood flow to the whole body due to the periodic contraction and relaxation of the heart, resulting in blood volume changes in vessels. Due to the different absorption and reflection capabilities of blood vessels and other tissues for different wavelengths of light, subtle color changes occur in skin areas with a rich vascular distribution, such as the face or palm. When a part of human skin tissue containing pulsatile blood is observed with a remote color camera, the camera measured signal of the skin surface would have a certain color variation over time, both due to the motion-induced intensity/specular variations and pulse-induced subtle color changes (Wang et al., 2017). Instead of the specular variations, the diffuse reflection component is associated with the absorption and scattering of light in skin tissues, which contains the pulse signal.

The task of rPPG algorithms is to derive the pulse signal from the RGB signals captured by the camera. Blazek et al. (Blazek et al., 2000) proved that blood pulse signals could be measured with a remote near-infrared imaging system. A similar technique was presented shortly after using a visual band camera (Wu et al., 2000). This concept was further developed by successful replications of this work in (Verkruysse et al., 2008). Verkruysse et al. first proved the feasibility of using a low-cost camera to detect the human heart rate (Verkruysse et al., 2008), and obtained the heart rate signal by analyzing a facial video taken under visible light. Many subsequent studies began to pay attention to artifact elimination during rPPG measurements, such as movement, facial expression, skin tone, illumination variations, etc.

In terms of eliminating the illumination artifacts, there are mainly two solutions: one is to directly separate the light change signal from the pulse signal through signal separation methods, and the other is to consider the non-skin background area except the face area as the artifacts reference (Nowara et al., 2020). Anti-motion interference methods can be roughly divided into 1) blind source analysis methods that separate the components of motion signals (Poh et al., 2010), 2) methods based on color models, such as CHROM (De Haan and Jeanne, 2013), POS (Wang et al., 2017), etc., which distinguish motion signals from pulse signals by analyzing skin color models, 3) methods based on motion compensation that include global and local motion compensation to eliminate the influence of head translation and rotation (Cheng et al., 2016).

However, it was found in practical applications that the signal separation-based methods can only aim at a specific interference, and cannot effectively deal with the coexistence of multiple interferences in a real scene. To further improve the robustness of non-contact rPPG pulse wave recovery, and also to explore the feasibility of rPPG recovery method based on deep learning, the research trend has changed from signal separation-based methods to data-driven methods.

### 2.2 Data-driven rPPG

The widespread use of deep learning in computer vision has led to the development of numerous contactless heart rate measuring techniques. Chen et al. proposed a convolutional attention network that employed normalized difference frames as input to predict the derivative of the pulse wave signal (Chen and McDuff, 2018). Niu et al. generated spatio-temporal map representation by aggregating information in multiple small regions of interest, and the spatio-temporal map was cascaded with ResNet to predict the heart rate (Niu et al., 2019a). Yu et al. designed the spatiotemporal networks PhysNet (Yu et al., 2020a) and rPPGNet (Yu et al., 2019) for pulse wave signal recovery, introduced the temporal difference information into the ordinary three-dimensional convolution network, and subsequently constrained the convergence of the model with a self-defined loss function (Yu et al., 2020b). Nowara et al. (2020) used the inverse operation of the attention mask to estimate the artifacts, and used it as the input of sequence learning to improve the estimation.

Transformer is becoming the preferred architecture for many computer vision tasks. By fully utilizing the self-attention mechanism to break through the space limitation of convolution computing, two recent rPPG works preliminarily showed that the transformer structure could match performance with the most advanced convolution network (Yu et al., 2022). However, whether it can exceed the performance of convolution network on large data sets remains to be studied (Kwasniewska et al., 2021). More recent works explore Transformer architecture with multimodal sources (e.g., RGB and NIR) (Liu et al., 2023), different color spaces (Liu et al., 2024), as well as multistage framework (Zhang et al., 2023; Zou et al., 2024a). Furthermore, a very recent sequence model backbone Mamba was also been investigated in the rPPG task (Zou et al., 2024b).

In addition, the training model based on generative adversarial network can generate realistic rPPG waveforms. For example, PulseGAN (Song et al., 2021) used conditional generative adversarial network to optimize the waveforms obtained with signal separation methods. Dual-GAN (Lu et al., 2021) used dual generative adversarial networks to model the background artifacts for better pulse wave recovery. However, this method involved facial landmark detection, ROI extraction, color space transformation, and other preprocessing. The complexity of preprocessing steps limits the real-time application in natural scenes.

In addition, there have also been attempts to use meta-learning methods (Lee et al., 2020). Liu et al. (2020) proposed a meta-learning framework, which used model-agnostic meta-learning algorithms for learning, and utilized signal separation-based methods to generate pseudo labels. However, due to the limitations of supervised learning, the performance of existing methods in cross databases evaluation and practical applications would be degraded. The learned spatiotemporal features are still vulnerable to lighting conditions and movements, and are unable to fully exploit the extensive temporal context to improve spatiotemporal representation. Therefore, introducing an enhanced temporal feature learning module might be a workable solution.

### 2.3 Spatio-temporal modeling

The rPPG signal contains temporal information that changes with the cardiac cycle, therefore, the modeling of spatio-temporal information is crucial. Early spatiotemporal deep learning networks directly extracted motion characteristics between frames using 3D convolution. Tran et al. (2015) suggested a homogeneous, small-scale C3D neural network to replace 3D convolution. Christoph et al. proposed a network called SlowFast, which consisted of a slow pathway that executed at a low frame rate and a fast pathway that executed at a high frame rate (Feichtenhofer et al., 2019). Liu et al. (2020) introduced a temporal shift module into the convolution network for rPPG-based physiological measurements on a mobile platform. Ouzar et al. (2023) end-to-end pulse rate estimation method based on depthwise separable convolutions.

The 3D-CDC (Yu et al., 2021; Yu et al., 2020c) was proposed as an innovative approach to replace 3D CNN. It was realized by a unified 3D convolution operator that incorporated spatio-temporal gradient information to deliver a more robust and discriminative modeling capability. The CDC has been adapted for tasks such as gesture recognition and face anti-spoofing, and has achieved the state-of-the-art performance. Yu et al. (2021) combined 3D-CDC with neural architecture search to perform gesture recognition. The difference between the aforementioned work and our work described in this paper is that the former uses 3D-CDC as a searchable convolution component. The neural architecture search usually needs large databases to support the search; however, for rPPG tasks, only relatively small data sets are available. Therefore, in this paper, the 3D-CDC module is directly applied to extract spatio-temporal representation, and subsequently combined with a dual branch structure and a soft attention mask to further take advantage of the rPPG-intrinsic temporal characteristics.

## 3 Methods

### 3.1 General framework

**FIGURE 1:**
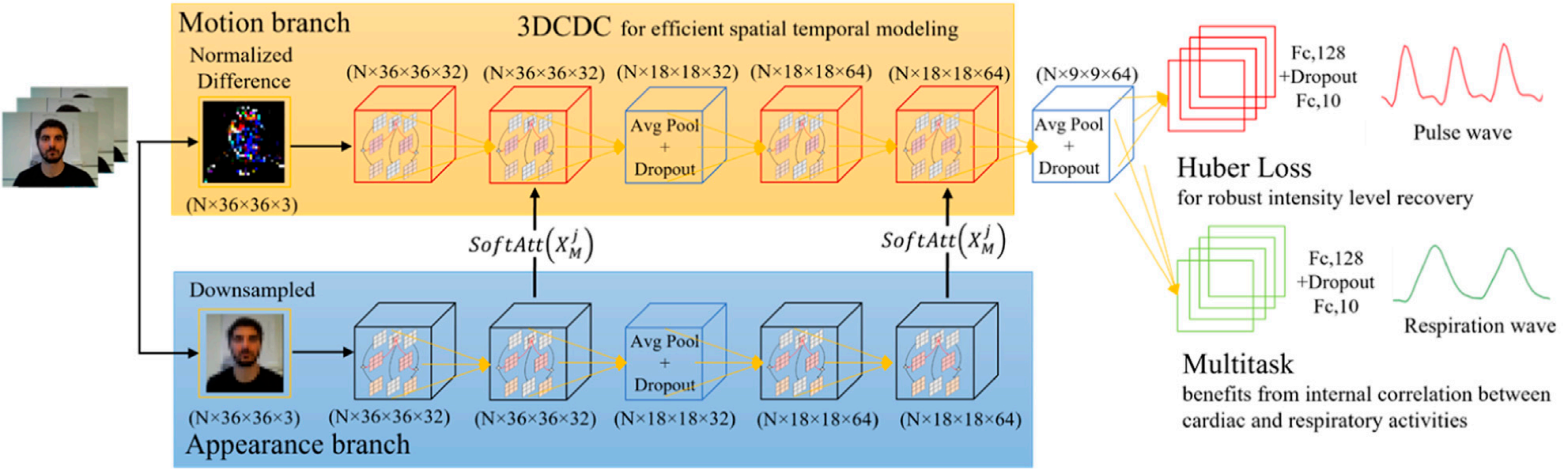
A multi-task 3D temporal central difference convolutional attention network for remote physiological measurements.

As Figure 1 shows, a multi-task 3D temporal central difference convolutional attention network with Huber loss was proposed to achieve robust pulse and respiration wave recovery. In particular, the normalized video frame difference is used as the input for motion representation based on the optical model of skin reflection, and a separate appearance branch is introduced to assign higher weights to the skin regions with stronger physiological signals. Temporal 3D-CDC is adapted as the backbone to capture rich temporal context. Multi-task measurement variant with Huber loss is then used to output the final prediction. The preprocessing of regions of interest extraction is not required in the proposed framework. The attention mechanism between two branches is deployed to achieve a similar function. As the distribution of physiological signals is not uniform in the whole facial area, the attention mechanism can learn soft-attention masks and assign higher weights to skin areas with stronger signals, which is beneficial for accuracy improvement.

### 3.2 Architecture

#### 3.2.1 Skin reflection modeling

The principle of rPPG is that when the light source irradiates skin tissues, the reflected light intensity would change with the variation of the measured substance (Wang et al., 2017). The measured substance here refers to the variation of blood volume in the blood vessel. The transmitted light intensity detected by the camera contains the corresponding physiological information of the tissue. Specifically, the skin reflections can be modeled as follows:

$$V_{k}\left(t\right)=I\left(t\right)\cdot \left(V_{s}\left(t\right)+V_{d}\left(t\right)\right)+V_{n}\left(t\right)$$ (1)

where $V_{k}\left(t\right)$ is the RGB value of the k^th^ skin pixel, $I\left(t\right)$ is the light intensity related to the light source, camera distance, and skin tissue absorption, $V_{n}\left(t\right)$ is the random quantization noise of the camera, and $V_{s}\left(t\right)$ and $V_{d}\left(t\right)$ represent the specular reflection and diffuse reflection of the skin, respectively. These reflections contain stationary and time-varying components. After expanding the two components given in Equation 1, $V_{k}\left(t\right)$ can be rewritten as

$$\begin{array}{c} V_{k}\left(t\right)=I_{0}\left(1+\underset{Intensity\;varaition}{\underbrace{\Psi \left(NP\left(t\right),P\left(t\right)\right)}}\right)\cdot \\ \left(\underset{Constant}{\underbrace{C}}+\underset{specular\;componets}{\underbrace{\Phi_{s}\left(NP\left(t\right),P\left(t\right)\right)}}+\underset{diffuse\;componets}{\underbrace{P\left(t\right)}}\right)+V_{n}\left(t\right) \end{array}$$ (2)

where $I_{0}$ denotes the static component of light intensity, $\Psi \left(NP\left(t\right),P\left(t\right)\right)$ is the intensity variation detected by the camera, and $\Phi_{s}\left(NP\left(t\right),P\left(t\right)\right)$ is the time-varying part of the specular reflection. The desired pulse wave signal is indicated by $P\left(t\right)$ , and $NP\left(t\right)$ indicates variations caused by non-physiological changes, such as changes in background light, head movement, speech, facial expression, etc.

The aim of any rPPG-based method is to extract $P\left(t\right)$ from $V_{k}\left(t\right)$ . To further simply Equation 2, spatial averaging of pixels is first applied to reduce the camera quantization error $V_{n}\left(t\right)$ . This is accomplished by using bicubic interpolation to downscale each frame to L-by-L pixels. The choice of L involves making a trade-off between reducing the camera noise and maintaining spatial resolution (Wang et al., 2015). Subsequently, any product between varying terms, such as $\Psi \left(NP\left(t\right),P\left(t\right)\right)\cdot P\left(t\right)$ , is neglected because the fixed components are significantly larger than the time-varying components. Furthermore, the constant term varies based on the subjects’ skin tone and lighting conditions, and is usually dominant, which can be reduced by taking the first order derivative of Equation 2 on both sides. After applying the above simplifications, we obtain

$$\begin{array}{c} V_{k}^{\prime }\left(t\right)=I_{0}\cdot \frac{\partial \Psi \left(NP\left(t\right),P\left(t\right)\right)}{\partial t}+I_{0}\cdot C\cdot \frac{\partial \Phi_{s}\left(NP\left(t\right),P\left(t\right)\right)}{\partial t}\\ +I_{0}\cdot \frac{\partial P\left(t\right)}{\partial t} \end{array}$$ (3)

It can be gathered from Equation 3 that $V_{k}\left(t\right)$ still depends on the observed stationary light intensity $I_{0}$ . The spatial distribution of $I_{0}$ is irrelevant to physiology, but is different in different video recording setups due to different distances to the light source and uneven skin contours (Chen and McDuff, 2018). The intensity $I_{0}$ can be removed by dividing $V_{k}^{\prime }\left(t\right)$ by the temporal mean of $V_{k}\left(t\right)$ as

$$\frac{V_{k}^{\prime }\left(t\right)}{\bar{V_{k}\left(t\right)}}=\frac{1}{C}\cdot \frac{\partial \Psi \left(NP\left(t\right),P\left(t\right)\right)}{\partial t}+\frac{\partial \Phi_{s}\left(NP\left(t\right),P\left(t\right)\right)}{\partial t}+\frac{1}{C}\cdot \frac{\partial P\left(t\right)}{\partial t}$$ (4)

Following (Chen and McDuff, 2018), the discrete-approximation form of Equation 4 can be written as

$$\frac{V_{k}^{\prime }\left(t\right)}{\bar{V_{k}\left(t\right)}}\approx \frac{V_{k}\left(t+∆t\right)-V_{k}\left(t\right)}{V_{k}\left(t+∆t\right)+V_{k}\left(t\right)}$$ (5)

which is the normalized frame difference and ∆t is the sampling interval.

Based on these deductions, a machine learning model would be suitable to capture the complex relationship between $V_{k}\left(t\right)$ and $P\left(t\right)$ . The normalized difference between consecutive frames can serve as the input of the motion branch of the learning model as illustrated in Equation 5. The motion representation thereby captures the physiological processes in a variety of lighting conditions. Subsequently, the appearance information in facial videos can be used to guide where and how the physiological processes should be approximated.

#### 3.2.2 Efficient spatio-temporal modeling

The rPPG is a periodic time-varying signal and, therefore, the spatio-temporal representation of facial video is the core step in rPPG signal extraction. The 3D convolution can naturally be used as a spatio-temporal information extractor. Compared with conventional 3D convolution, temporal 3D-CDC concentrates on the differences in temporal gradient by including the temporal gradient data into a single 3D convolution operation. This results in calculation of the central difference from the adjacent local spatio-temporal region (Yu et al., 2020d). The 3D-CDC contains two main steps with a tendency to converge towards the center-oriented temporal gradient of the sampled values, which can be expressed as Equation 6:

$$3DCDC\left(l_{0}\right)=\sum_{l_{n}\in C}\omega \left(l_{n}\right)\cdot x\left(l_{0}+l_{n}\right)+\theta \cdot \left(-x\left(l_{0}\right)\cdot \sum_{l_{n}\in R}\omega \left(l_{n}\right)\right)$$ (6)

where x is the input feature map, C denotes the local receptive field cube, ω are the learnable weights, $l_{0}$ represents the current location on the feature map, and $l_{n}$ enumerates the locations in C and adjacent time steps in R . The hyperparameter θ tradeoffs the importance of intensity and gradient information. The 3D-CDC can provide a more discriminative and reliable modeling capability without any extra parameters.

#### 3.2.3 Dual branch and soft attention

The first order derivative during the reflection modeling is used to remove the constant terms that are generally associated with the subjects’ skin tone and lighting conditions. The proposed model could partially reduce the dependence of the learned model on skin tones and lamp spectra in the training data. In the motion representation, however, each pixel is assumed to be equally weighted in skin reflection modeling. Although the use of normalized frame difference helps to reduce the influence of background pixels to a certain extent, it would still cause an increase in artifacts and affect the rPPG measurement. Previous studies have used custom regions of interest for rPPG measurement. However, this usage requires additional preprocessing such as facial landmark detection or skin detection. Not all skin pixels contribute equally to rPPG measurement because physiological signals are not evenly distributed in skin regions. Therefore, it would be beneficial to add an attention module to assign a higher weight to skin areas with a stronger physiological signal representation.

As the differential operation in the motion representation process removes the appearance information, a separate appearance branch is utilized based on (Chen and McDuff, 2018). Unlike the motion branch, which uses the normalized frame differences as the input, the downsampled frame is considered as the input of the appearance branch. The two branches have the same structure except for the lack of last three layers in the appearance branch. The attention masks could be estimated with a 1 × 1 × 1 convolution filter right before the pooling layers. The soft attention mask is defined in Equation 7:

$$SoftAtt\left(X_{M}^{j}\right)=\frac{S\left(\omega^{j}X_{A}^{j}+b^{j}\right)\cdot H^{j}\cdot W^{j}}{2\left\|S\left(\omega^{j}X_{A}^{j}+b^{j}\right)\right\|}⊙X_{M}^{j}$$ (7)

where $X_{A}^{j}$ and $X_{M}^{j}$ are the feature maps of the convolution layer j of the appearance and motion branches, respectively, and $H^{j}$ and $W^{j}$ are the height and width of the feature maps of the convolution layer j , respectively. The sigmoid function is denoted by $S\left(\cdot \right)$ , $\omega^{j}$ and $b^{j}$ are the weights and bias of the convolution kernel, respectively, $\left\|\cdot \right\|$ is the $l_{1}$ norm, and ⊙ denotes the element-wise product. The soft attention mask is obtained by the sigmoid function followed by L1 normalization, which generates a soft attention mask that can avoid extreme values. The attention mask is the bridge between the motion and appearance branches to assign higher weights to the skin regions with stronger physiological signals through joint learning.

#### 3.2.4 Multi-task and loss function

There are generally two types of loss functions widely used in deep learning-based rPPG. One is the loss function that aims to minimize the point-by-point error, such as the MAE and RMSE. The other one minimizes the waveform similarity error, such as the negative Pearson correlation coefficient. The former focuses on the intensity level constraint, which is relatively simple and easy to converge, but may cause overfitting. For instance, the MSE would decrease slowly to approach the local minimum during gradient descent. However, as the RMSE is a squared error, it would be sensitive to abnormal values. The MAE can reduce the sensitivity to outliers, but its gradient remains relatively constant that may miss the local minimum. In contrast, the latter constraint is in the frequency domain, forcing the model to learn periodic features in the target frequency band. As the artifacts in rPPG may be large in a realistic environment, these losses would be difficult to converge. Therefore, we compare different loss functions (MAE, RMSE, negative Pearson, ε-insensitive, and their combinations, detailed in Section 5.4), and find that the Huber loss achieves the best rPPG recovery performance. The Huber loss equation is as follows:

$$L_{huber}=\frac{1}{N}\sum_{i=1}^{N}\;I_{|y_{i}-{\hat{y}}_{i}|\leq \delta }\frac{{\left(y_{i}-{\hat{y}}_{i}\right)}^{2}}{2}+I_{|y_{i}-{\hat{y}}_{i}|>\delta }\left(\delta |y_{i}-{\hat{y}}_{i}|-\frac{1}{2}\delta^{2}\right)$$ (8)

where $y_{i}$ is the ground truth pulse waveform or respiration waveform and ${\hat{y}}_{i}$ is the respective predictions by the proposed method. When the error between the predicted rPPG signal and the ground-truth is less than or equal to the threshold δ (default value 1 is used), the loss function degenerates from Huber to RMSE; otherwise, it degenerates from Huber to MAE. Thus, the Huber loss can combine the advantages of RMSE and MAE while being less sensitive to outliers in the training data.

Due to respiratory sinus arrhythmia, which is a rhythmic fluctuation of the cardiac cycle in the respiratory frequency, the PPG signals also include information about respiration (Berntson et al., 1993). Thus, the physiological signal $P\left(t\right)$ is an intricate synthesis of the pulse and respiration waves. These two are related in terms of the underlying mechanism. Therefore, a multi-task network is constructed to measure pulse and respiratory signals simultaneously, reducing the computational cost by about half. The intermediate representation can be shared, and only different fully connected layers are used to regress pulse and respiration separately, as shown in Figure 1. The multi-task loss is defined as

$$L^{Total}=\alpha \cdot L_{Huber}^{hr}+\beta \cdot L_{Huber}^{rsp}$$ (9)

where α=β=1 is adapted in our experiment based on empirical studies.

## 4 Results

### 4.1 Database and evaluation settings

The proposed method is evaluated on three publicly available databases: UBFC (Bobbia et al., 2019), PURE (Stricker et al., 2014), and COHFACE (Heusch et al., 2017), which are commonly used in recent research.

The UBFC-rPPG database (Bobbia et al., 2019) comprises 42 videos from 42 subjects recorded with a web camera at a rate of 30 frames per second, a resolution of 640 × 480, and stored in an uncompressed format. A pulse oximeter was used to obtain the ground-truth PPG data. All scenes were indoors in different lighting conditions. During collection, subjects were asked to do mental arithmetic as a manipulation of heart rate. The PURE database (Stricker et al., 2014) contains 10 subjects, each of whom participated under six different recording conditions, e.g., sitting still, speaking, slow/fast head movements, etc. This database was recorded at 30 frames per second using an industrial camera and stored in an uncompressed format with a resolution of 640 × 480. The PPG data were also collected by a pulse oximeter.

The COHFACE database (Heusch et al., 2017) comprises 160 videos from 40 subjects, where four videos involving each subject were taken with two different lighting conditions. The videos were recorded with a webcam at a rate of 20 frames per second, a resolution of 640 × 480, and compressed in MPEG-4 format. The bit rate was 250 kb/s, which made it considerably challenging due to the compression. The recorded physiological signals included blood volume pulse and respiratory signals. Note that other databases also exist for rPPG research, such as VIPL-HR (Niu et al., 2018), OBF (Li et al., 2018) and AFRL (Estepp et al., 2014). The OBF and AFRL databases are currently not publicly available. We obtained the VIPL-HR database and the ground-truth PPG signals for training our method. However, after further investigation, we found that the PPG signals in VIPL-HR were not evenly sampled. The ratio of sampling points of contact PPG to the frame number of videos varied from 2 to 4, making it not suitable for training our method.

The average heart rate (HR) estimation task is evaluated on all three databases while the respiration rate (RR) estimation task is evaluated on the COHFACE database. Particularly, we follow the evaluation settings in (Niu et al., 2020; Tsou et al., 2020; Heusch et al., 2017). For the UBFC database, data from 28 subjects are used as the training set and those from the remaining 14 subjects are used as the test set. For PURE and COHFACE databases, data from 60% of subjects are used for training, and those from the remaining 40% are used for testing.

### 4.2 Implementation details

To avoid overfitting, the second and fourth convolutional layers are followed by two average pooling layers and two dropout layers, respectively^1^. The input of the appearance branch is preprocessed by downsampling each video frame to a size of 36 × 36, since 36 is supposed to be the optimal value for retaining spatial resolution while reducing camera noise (Chen and McDuff, 2018). We pick α=β=1 for the multi-task loss function in order to force the pulse and respiration estimations to be regraded equally. A second-order Butterworth filter is used to further filter the network’s output. The cut-off frequencies for HR are 0.75 and 2.5 Hz, and for RR they are 0.08 and 0.5 Hz. The position of the highest peak in the power spectrum obtained from the filtered signal is used to determine the estimated HR or RR.

We implement our method in TensorFlow 2.0. Adadelta optimizer is used to train the model with an NVIDIA GeForce RTX 2080Ti GPU. The learning rate is set as 1.0, and all other parameters are the same as the default parameters of the Adadelta optimizer. The number of training epochs is chosen differently for different databases with early stopping based on visual inspection of ten-fold cross-validation. Apart from the proposed method with 3D-CDC, we also implement a standalone 3D CNN model to verify the effectiveness of the central difference mechanism. All other modules are the same except for the convolution operation. During HR-only evaluations, the network was trained based on a HR-based loss where α=1 and β=0 in Equation 9.

Another thing to note is that we do not reproduce all previous methods, but refer to the results from the corresponding papers. For comparison, classical non-deep learning methods, such as POS (Wang et al., 2017) and CHROM (De Haan and Jeanne, 2013) are used as a baseline. Not all previous methods are evaluated on the aforementioned three databases; however, the state-of-the-art methods in each database are compared, such as Dual-GAN (Lu et al., 2021) in UBFC, DeepPhys (Chen and McDuff, 2018) in PURE, and DeeprPPG (Liu and Yuen, 2020) in COHFACE.

### 4.3 Intra-database evaluation

#### 4.3.1 HR estimation on UBFC-rPPG

**FIGURE 2:**
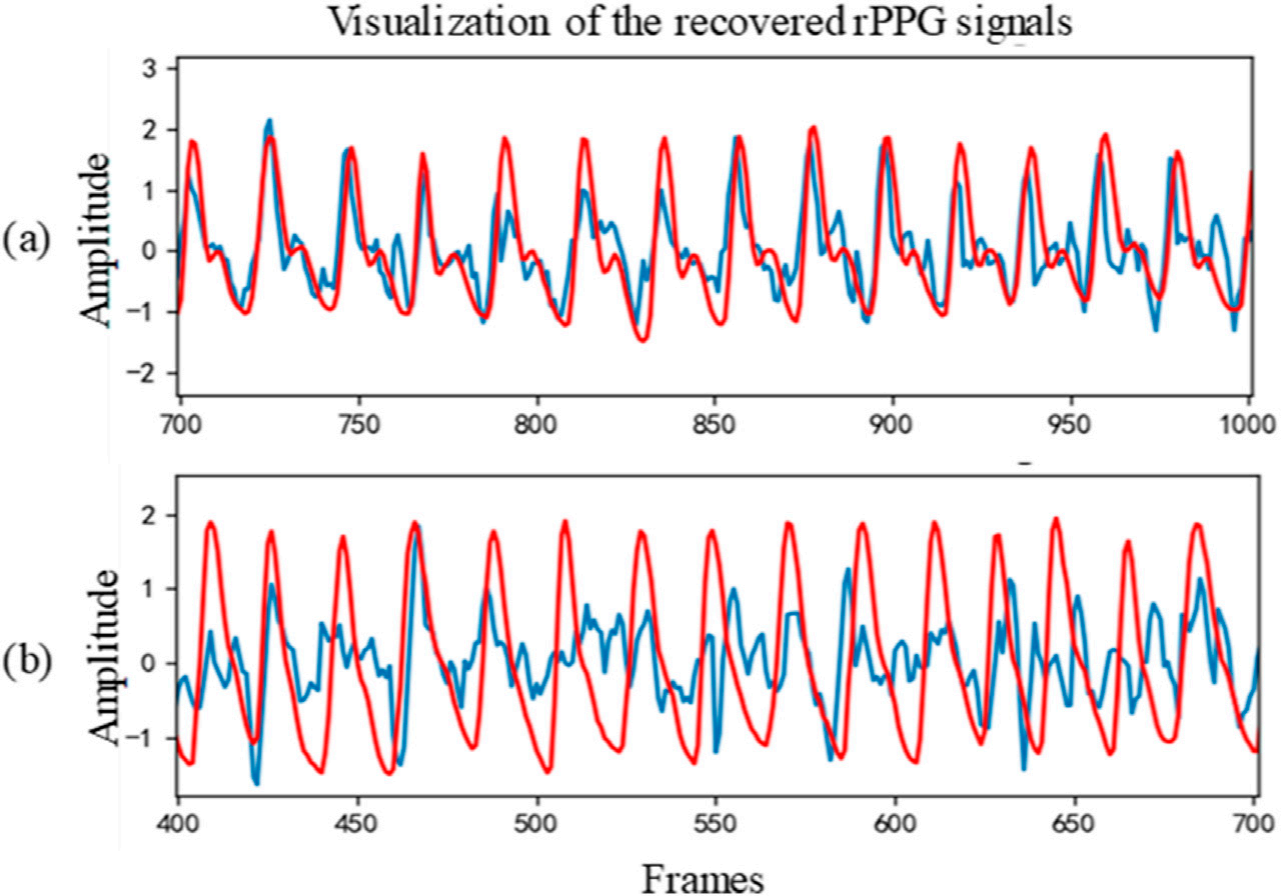
Two cases of the recovered signal curve on UBFC-rPPG: **(A)** Subject 39 and **(B)** Subject 48. The red line represents the recovered signal, while the blue line represents the ground-truth.

Table 1 shows the intra-database evaluation results on the UBFC-rPPG database. The results show that the proposed method based on 3D temporal central difference convolutional attention network outperforms both the traditional and recent deep learning-based methods with MAE, RMSE, and correlation coefficient of 0.34, 1.12, and 0.997 respectively. It is important to note that the evaluation metrics MAE and RMSE represent the MAE or RMSE of estimated heart rate and respiration rate, rather than the point-by-point error in loss functions. Two examples of the rPPG signal predicted by the proposed rPPG recovery network on this database and the corresponding ground-truth PPG signal collected by the sensor are shown in Figure 2. In most cases, the recovered curve fits with the ground-truth signals, but there are unfavorable cases such as the one shown in Figure 2B. The failure may be due to the noisy ground-truth signal, which can be caused by artefacts during sensor collection. Even under this noisy condition, our method is still able to reconstruct a sinusoidal-like curve.

**TABLE 1:** Intra-database evaluation on UBFC-rPPG.

UBFC	Year	MAE	RMSE	r
CHROM (De Haan and Jeanne, 2013)	2013	3.44	4.61	0.97
POS (Wang et al., 2017)	2017	2.44	6.61	0.94
MODEL (Li et al., 2019)	2019	3.99	5.55	0.75
MAICA (Macwan et al., 2019)	2019	3.34	—	0.72
CK (Song et al., 2020)	2020	2.30	3.80	0.98
ETA-rPPGNet (Hu et al., 2021)	2021	1.46	3.97	0.93
Dual-GAN (Lu et al., 2021)	2021	*0.44*	*0.67*	*0.99*
AND-rPPG (Lokendra and Puneet, 2022)	2022	2.67	4.07	0.96
PhysFormer (Yu et al., 2022)	2022	0.50	**0.71**	0.99
RhythmMamba (Zou et al., 2024b)	2024	0.50	0.75	0.99
OURS		**0.34**	1.12	**0.997**

Best results in Bold and second-best results in Italics.

#### 4.3.2 HR estimation on PURE

**FIGURE 3:**
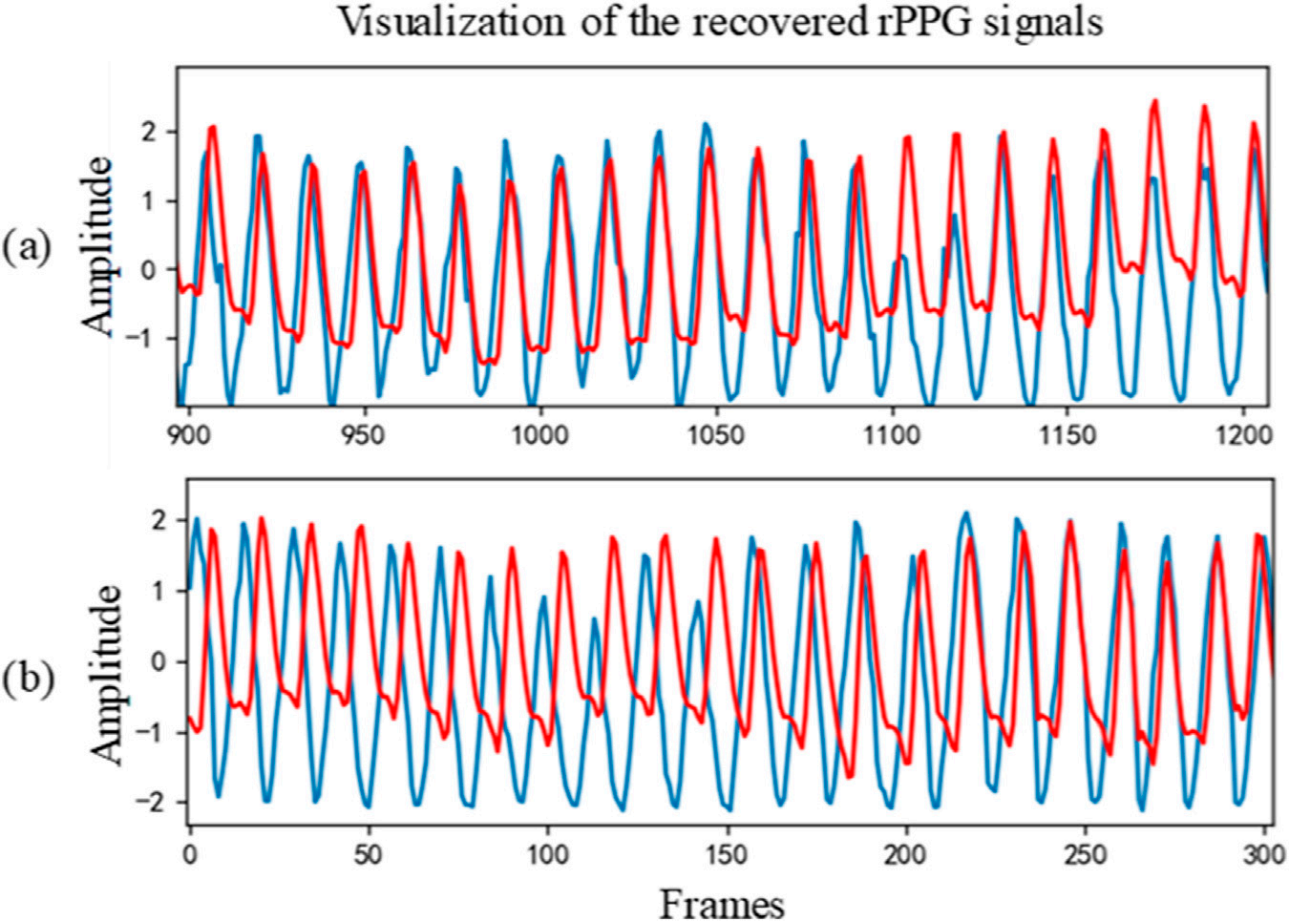
Two cases of recovered PPG signal curve on PURE: **(A)** Sample 07–03 and **(B)** Sample 07–01. The red line represents the recovered signal, while the blue line represents the ground-truth.

Table 2 shows the intra-database evaluation results on the PURE database. The results show that the proposed method outperforms existing methods with MAE, RMSE, and correlation coefficient of 0.78, 1.07, and 0.999, respectively. Two examples of the rPPG signal predicted on the PURE database and the corresponding ground-truth signal are also shown in Figure 3. Even for the “bad” cases in Figure 3B, the recovered curve generally fits well with the ground-truth, exhibiting a small phase difference.

**TABLE 2:** Intra-database evaluation on PURE.

PURE	Year	MAE	RMSE	r
CHROM (De Haan and Jeanne, 2013)	2013	2.07	2.50	*0.99*
LiCVPR (Li et al., 2014)	2014	28.2	30.96	−0.38
2SR (Wang et al., 2016)	2015	2.44	3.06	0.98
POS (Wang et al., 2017)	2017	3.14	10.57	0.95
HR-CNN (Špetlík et al., 2018)	2018	1.84	2.37	0.98
DeepPhys (Chen and McDuff, 2018)	2018	0.83	1.54	0.99
PhysNet (Yu et al., 2020a)	2020	1.90	3.44	0.98
Dual-GAN (Lu et al., 2021)	2021	*0.82*	*1.31*	*0.99*
MSDN (Zhang et al., 2023)	2023	1.46	1.96	0.99
OURS		**0.78**	**1.07**	**0.999**

Best results in Bold and second-best results in Italics.

#### 4.3.3 HR and RR estimation on COHFACE

**FIGURE 4:**
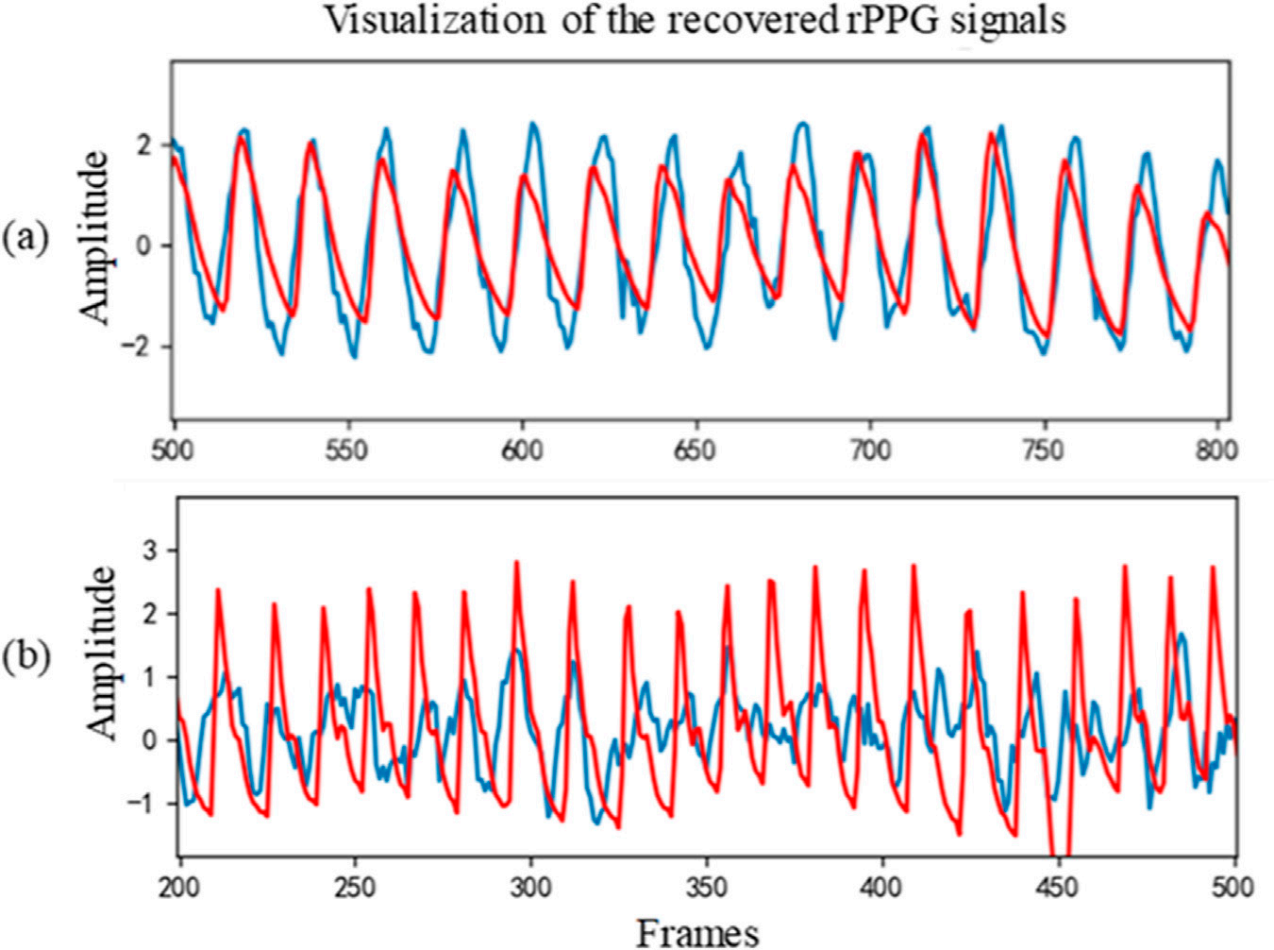
Two cases of recovered signal curve on COHFACE: **(A)** Sample 32–02 and **(B)** Sample 35–02. The red line represents the recovered signal, while the blue line represents the ground-truth.

**FIGURE 5:**
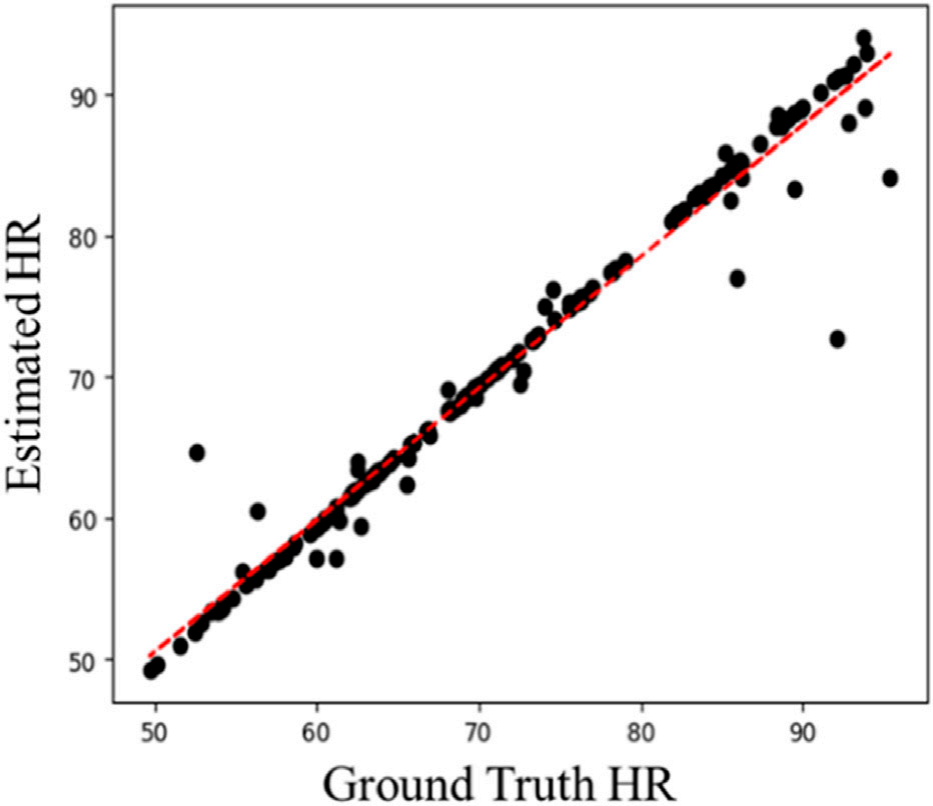
Scatter plot of estimated HR vs. ground-truth HR on COHFACE.

Table 3 shows the intra-database evaluation results on the COHFACE database. The results show that the proposed method significantly outperforms prior methods with MAE, RMSE, and correlation coefficient of 1.71, 3.57, and 0.965, respectively. The video compression of COHFACE does not perform as well as the other two databases. Two examples of the rPPG signal predicted by the proposed rPPG recovery network on the COHFACE database and the corresponding ground-truth signal are also shown in Figure 4. Again, the recovered curve does not properly fit the noisy ground-truth signal shown in Figure 4B. As the scatter plot shows in Figure 5, all points are clustered around the diagonal line, and only a few samples show deviation, where the underestimation of HR is higher compared to its overestimation. Scatter plots for UBFC-rPPG and PURE are not drawn because they are almost overlaid with the diagonal line due to the good estimation.

**TABLE 3:** Intra-database evaluation on COHFACE.

COHFACE	Year	MAE	RMSE	r
CHROM (De Haan and Jeanne, 2013)	2013	7.8	12.45	0.26
LiCVPR (Li et al., 2014)	2014	19.98	25.59	−0.44
2SR (Wang et al., 2016)	2015	20.89	25.84	−0.32
POS (Wang et al., 2017)	2017	13.43	17.05	0.07
HR-CNN (Špetlík et al., 2018)	2018	8.10	10.78	0.29
DeeprPPG (Liu and Yuen, 2020)	2020	*3.07*	7.06	*0.86*
ETA-rPPGNet (Hu et al., 2021)	2021	4.67	6.65	0.77
AND-rPPG (Lokendra and Puneet, 2022)	2022	3.82	*5.10*	0.79
MSDN (Zhang et al., 2023)	2023	3.87	4.69	0.81
OURS		**1.71**	**3.57**	**0.965**

Best results in Bold and second-best results in Italics.

**FIGURE 6:**
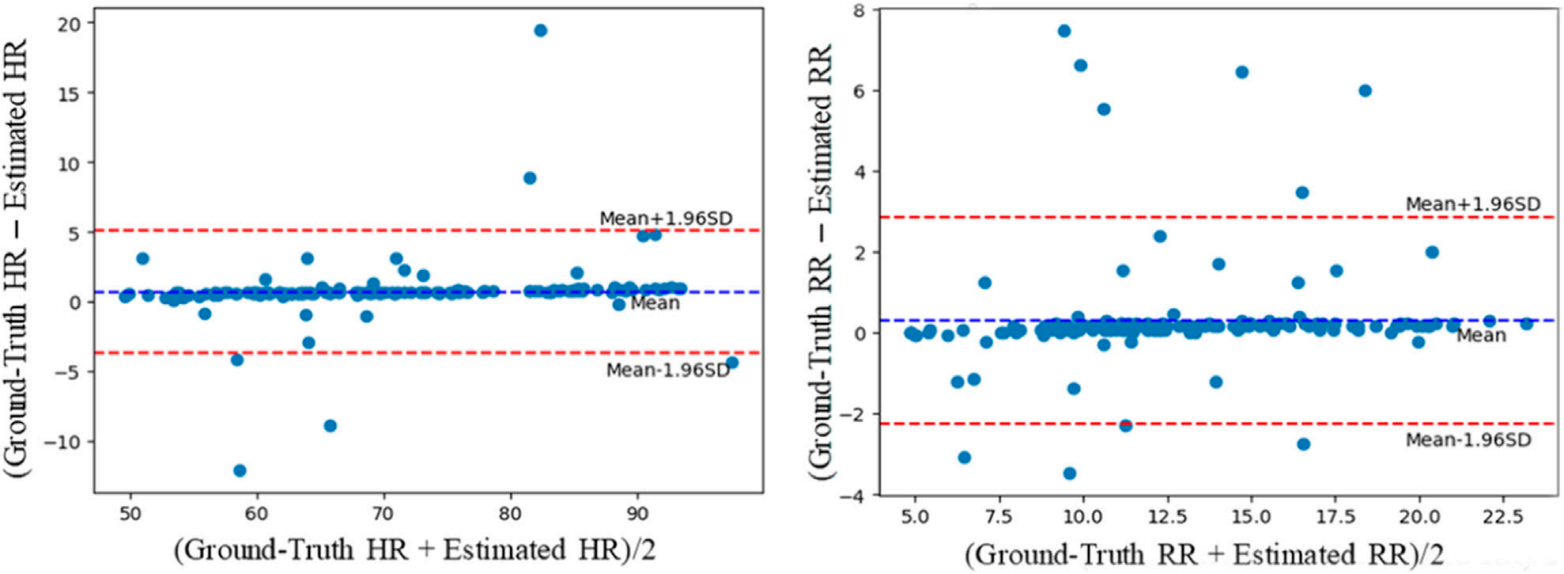
Bland-Altman plots evaluating the agreement between the ground-truth HRs and RRs and obtained by the contact sensor and HRs and RRs estimated by the proposed methods for the COHFACE database.

As stated in Section 4.1, the COHFACE database contains not only pulse signals but also the respiratory signals. The multi-task setting (Section 3.2) is evaluated on this database. There are relatively few studies on RR estimation. Two previous methods and one multi-task 3D convolution method are compared. As Table 4 shows, the proposed multi-task method achieves good performance with MAE, RMSE, and correlation coefficient of 1.07, 2.41, and 0.80, respectively. The Bland-Altman plot is also used to assess the differences in measurements between the estimated results and the references. As Figure 6 shows, the *x*-axis is the mean value of the ground-truth HR (RR) and estimated HR (RR), and the *y*-axis is the deviation between the ground-truth HR (RR) and estimated HR (RR). Most of the values are distributed between mean ± 1.96SD, indicating that the results of HRs (RRs) measured by the model are close to those measured by the contact sensor.

**TABLE 4:** Multi-task network for heart rate and respiratory rate estimation on COHFACE.

	Heart rate			Respiratory rate		
Method	MAE	RMSE	r	MAE	RMSE	r
Liu et al. (2020)	4.27	—	—	5.73	—	—
Ren et al. (2021)	1.81	—	—	5.39	—	—
MT3D	1.61	3.08	0.975	2.57	4.53	0.34
OURS	1.53	2.92	0.978	1.07	2.41	0.80

### 4.4 Cross-databases evaluation

The above evaluations are carried out in an intra-database manner. It is relatively trivial for deep neural networks to train and converge within one database, but they usually show a poor generalization ability across different databases. We further conduct a cross-database evaluation to verify the effectiveness and generalization ability of the proposed method for scenes without any prior knowledge. There are usually large differences between different databases, such as ambient lighting, subject movements, acquisition apparatus differences, etc.

Consider PURE and COHFACE databases as examples: 1) The illumination conditions of the two databases are different during data acquisition. The subjects in PURE sit in front of the window, and the light changes with the movement of the cloud, while the data collection in COHFACE is carried out under two different lighting conditions: indoor light and natural light; 2) There are differences in the status of subjects at the time of data collection for different databases. The PURE database contains six different states, while the subjects in the COHFACE remain stationary without any head movement; 3) Different databases may use different cameras. The PURE database utilizes the industrial camera SVS vistek GmbH for data acquisition. It collects the data at the rate of 30 frames per second and stores them in the uncompressed format. The video in the COHFACE database is acquired using the Logitech c525 network camera to record at the rate of 20 frames per second. The H.264 compression format is utilized to compress at the rate of 250 bits per second. From the above analysis, it can be gathered that the two databases are considerably different in terms of lighting conditions, subject movement, and camera settings. It would be challenging to generalize across the two databases, which necessitates the evaluation of the generalization ability of the proposed method.

We first conduct the cross-database evaluation with training on PURE while testing on COHFACE. Table 5 shows the corresponding results. Compared with intra-database evaluation results in Table 3, the performance shown in Table 5 deteriorates as expected. Though the performance is worse than other deep neural network methods trained on COHFACE, the proposed method still performs better than the traditional methods such as POS, CHROM, LiCVPR, 2SR, etc. We further evaluate training on COHFACE and testing on PURE, and the corresponding results are shown in Table 6. The experimental results are MAE = 1.73, RMSE = 2.87, and r = 0.942. These results are comparable to the intra-database testing results shown in Table 2. Overall, the cross-database evaluation further demonstrates that the proposed rPPG measurement network has good generalization ability for unknown scenes.

**TABLE 5:** Cross-database evaluation: trained on PURE, tested on COHFACE.

PURE→COHFACE	MAE	RMSE	r
OURS-CrossDatabase	6.65	12.38	0.476

**TABLE 6:** Cross-database evaluation: trained on COHFACE, tested on PURE.

COHFACE→PURE	MAE	RMSE	r
OURS-CrossDatabase	1.73	2.87	0.942

### 4.5 Impact of video compression

Previous studies have shown that video compression significantly impacts rPPG recovery: the signal-to-noise ratio drops dramatically as the video bitrate increases (Nowara et al., 2021). In the three public databases used in this paper, the videos in UBFC-rPPG and PURE are stored in an uncompressed format, while the videos in COHFACE are stored in the H.264 compressed format with a bit rate of 250 kb/s. Video data stored in uncompressed format require larger storage space that makes it difficult to analyze, operate and share the videos. For example, the storage space required for a one-minute video in UBFC-rPPG is about 1.7 GB, which is about 936 times the storage space required for the same length video in COHFACE. Video compression algorithms help in saving storage space, with the aim of reducing the bit rate of video while maintaining the perceptual visual appearance. Compression algorithms eliminate subtle changes in intensity between frames that have a minor impact on visual appearance. However, the rPPG measurement relies on these subtle intensity and color changes to measure the physiological signals, which makes it difficult to recover physiological information from compressed videos. Therefore, the research on the effect of video compression on rPPG measurements is of great significance for practical applications.

In this section, we evaluate the extent to which the proposed rPPG network can resist the impact of video compression. In particular, the original videos from PURE database are compressed in the format of H.264 with a bit rate of 250 kb/s, and the experiment results before and after compression are compared. Following (Yu et al., 2019), FFmpeg is used as the compression tool. Under this compression rate, the required storage space for a one-minute-length video in PURE is reduced from 890 MB to 2 MB. Table 7 shows the evaluation results. The results of all rPPG recovery methods on PURE database are significantly inferior compared to those on the uncompressed version.

**TABLE 7:** Evaluation of video compression on rPPG measurement (results on PURE).

Compression	Pre			After			Percentage of decrease		
Methods	MAE	RMSE	r	MAE	RMSE	r	MAE	RMSE	r
CHROM (De Haan and Jeanne, 2013)	2.07	2.50	0.99	6.29	11.36	0.55	−2.04	−3.54	−0.44
2SR (Wang et al., 2016)	2.44	3.06	0.98	5.78	12.81	0.98	−1.37	−3.19	−0.00
HR-CNN (Špetlík et al., 2018)	1.84	2.37	0.98	8.72	11.00	−0.7	−3.74	−3.64	−1.71
PhysNet (Yu et al., 2020a)	1.90	3.44	0.98	5.39	11.05	0.76	−1.84	−2.21	−0.22
3DCDC-T	0.78	1.07	0.99	1.72	3.99	0.89	−1.21	−2.73	0.10

### 4.6 Evaluation of loss function

We compare the performance of the proposed method based on spatio-temporal efficient modeling while using different loss functions, including MAE, RMSE and negative Pearson correlation coefficient that are commonly deployed in rPPG measurements. In addition, to combine the advantages of RMSE and MAE as loss functions and avoid the disadvantages of using them separately, we also evaluate the Huber loss, ε-insensitive Huber loss, and a combination of Huber loss and Pearson correlation coefficient. Specially, the ε-insensitive loss function is commonly used in support vector regression and can be formulated by Equation 10:

$$L_{\varepsilon }\left(y,f\left(x\right)\right)=\max\left(0,\left|y-f\left(x\right)\right|-\varepsilon \right)$$ (10)

which does not penalize the samples whose error is less than or equal to ε. Its aim is to ignore small noisy training samples in insensitive areas (Wang et al., 2022). After combining with the Huber loss function in Equation 8, the ε-insensitive Huber loss can be expressed as Equation 11:

$$L\left(y,f\left(x\right)\right)=\left\{\begin{array}{ll} 0 & 0\leq \left|y-f\left(x\right)\right|\leq \varepsilon \\ {\frac{1}{2}\left(\left|y-f\left(x\right)\right|-\varepsilon \right)}^{2} & \varepsilon <\left|y-f\left(x\right)\right|\leq \delta +\varepsilon \\ \delta \left(\left|y-f\left(x\right)\right|-\varepsilon \right)-{\frac{1}{2}\delta }^{2} & \delta +\varepsilon <\left|y-f\left(x\right)\right| \end{array}\right.$$ (11)

We evaluate the ε-insensitive Huber loss function with a default value of ε = 0.1. Increasing this value further may degrade accuracy (Lambert et al., 2022). As Table 8 shows, the Huber loss consistently achieves the best results for three metrics on two databases. The ε-insensitive Huber loss performs marginally worse compared to the Huber loss may because the former loss function makes the model focus on samples with large prediction errors, which may sacrifice the accuracy (Balasundaram and Prasad, 2020).

**TABLE 8:** Evaluation of different loss functions on COHFACE and UBFC.

	UBFC			COHFACE		
	MAE	RMSE	r	MAE	RMSE	r
MAE	0.99	2.42	0.988	1.86	4.02	0.955
RMSE	0.80	1.99	0.992	1.95	4.28	0.946
Huber	**0.34**	**1.12**	**0.997**	**1.71**	**3.57**	**0.965**
Negative Pearson	0.80	1.99	0.993	1.74	4.12	0.953
Huber + Pearson	0.80	1.96	0.992	1.88	4.26	0.948
ε -insensitive Huber loss	0.53	1.65	0.995	1.90	4.89	0.934

Best results in Bold.

### 4.7 Ablation study

In the ablation study, we remove one module (e.g., 3D-CDC, the soft attention, and multi-task setting) each time to see the effect of that module on the performance.

#### 4.7.1 Impact of 3D-CDC module

In this ablation, we consider the configurations with or without the CDC module. It can be observed from the last row in Table 9 that the CDC module helps the proposed method perform better, exhibiting an increase in MAE, RMSE, and r on all three databases.

**TABLE 9:** Ablation of central difference convolution module.

	UBFC			PURE			COHFACE		
	MAE	RMSE	r	MAE	RMSE	r	MAE	RMSE	r
3D	0.83	1.99	0.992	1.26	2.14	0.998	1.87	3.90	0.960
With CDC module	0.34	1.12	0.997	0.78	1.07	0.999	1.71	3.57	0.965

#### 4.7.2 Impact of soft attention

In order to verify the effectiveness of the dual branch structure, we conduct an ablation study of the attention module. Table 10 lists the results. The incorporation of the attention module shows consistent improvements among the three databases, which indicates that the attention mask learned from the appearance branch assists motion representation.

**TABLE 10:** Ablation of soft attention module.

	UBFC			PURE			COHFACE		
	MAE	RMSE	r	MAE	RMSE	r	MAE	RMSE	r
w/o attention module	1.05	2.41	0.986	1.53	4.28	0.940	2.73	5.09	0.908
w. attention module	0.34	1.12	0.997	0.78	1.07	0.999	1.71	3.57	0.965

#### 4.7.3 Impact of multi-task setting

Table 11 shows the ablation of multi-task settings. It can be observed that these settings not only realize the RR prediction, but also improve the heart rate estimation results. The two physiological signals are believed to be correlated. The multi-task architecture may benefit from this internal correlation that results in an improved performance both on HR and RR.

**TABLE 11:** Ablation of multi-task setting.

	Heart rate			Respiratory rate		
Method	MAE	RMSE	r	MAE	RMSE	r
Single-Task	1.71	3.57	0.965	—	—	—
Multi-Task	1.53	2.92	0.978	1.07	2.41	0.80

## 5 Discussion

The above results show the effectiveness of the proposed method, which achieves the best or second-best results on all three databases (shown in Tables 1–3). In contrast with the previous state-of-the-art methods, the proposed method is generally distinct from the other deep learning-based methods from two aspects: One is the spatio-temporal network. For instances, DeepPhys (Yu et al., 2020a) and PhysNet (Chen and McDuff, 2018) employ 3D CNN, while MTTS (Liu et al., 2020) adapts the temporal shift modules that aim to reduce the computational budget without any accuracy gain. The 3D-CDC utilized in this paper can replace conventional convolutional operations without extra parameters.

One of the possible reasons for the improvements in results is the suitability of the enhanced spatio-temporal context modeling ability to represent the appearance and motion information. In the meantime, the central difference component can be considered as a regularization term to alleviate overfitting (Yu et al., 2021). We would assume that the emphasis on simulating the temporal component of the physiological signals would increase the resistance to artifacts.

The second aspect is the network architecture, such as Dual-GAN (Lu et al., 2021), which is an elegant design of GAN-based architecture for signal disentanglement, and performs better than our method on some metrics, such as RMSE on UBFC-rPPG. However, Dual-GAN contains the pre-processing step called the spatio-temporal map generation. This requires preprocessing operation including face detection, facial landmarks localization, face alignment, skin segmentation, and color space transformation, which are considerably complicated. On the other hand, our proposed method only needs a simple subtraction operation between frames as the input for the motion branch. During cross-database evaluation, one possible reason for the relatively good performance of training on COHFACE is compression. Deep learning-based methods perform well on uncompressed data where the model has seen compressed samples during training, but not vice versa. A similar pattern has also been reported in a recent study (Nowara et al., 2021).

Furthermore, to help us understand the reason for the effectiveness of the proposed method, we also evaluate the effects of loss functions and video compression, and conduct the ablation study. When the loss between the recovered rPPG and ground-truth signals approaches the minimum value, the gradient decreases slowly with Huber loss. Consequently, the model would be more robust for rPPG signals prediction. We also observe that the multi-task variant provides an accuracy improvement relative to the single-task versions because the network may be able to simultaneously model internal relevance and save computational budget. The proposed method seems less affected by video compression compared with other methods. This verifies that although video compression impacts rPPG measurements, the proposed network with efficient spatio-temporal modeling is robust against the impact of video compression to a certain extent. Overall, the rPPG measurement network based on efficient spatio-temporal modeling can capture rich temporal context by aggregating rPPG-related temporal difference information.

Contactless measurement technologies like remote photoplethysmography (rPPG) are gaining traction in the clinical field due to their potential to revolutionize patient monitoring. For instance, 1) rPPG allows for the measurement of vital signs without physical contact, which is especially beneficial in environments where patient comfort is paramount, such as neonatal care, burn units, or for individuals with sensitive skin; 2) rPPG can be integrated into telehealth platforms, allowing for real-time physiological monitoring during virtual consultations; 3) Contactless methods reduce the risk of cross-contamination, which is particularly relevant in pandemic situations such as COVID-19, where minimizing direct contact between patients and healthcare workers became a top priority. This advantage makes rPPG an attractive option in infectious disease wards or intensive care units. However, despite its initial success, the application of rPPG in clinical settings faces several challenges and limitations. For example, data privacy and ethical considerations. Since rPPG involves the use of cameras, there are concerns related to privacy, especially in clinical and home settings. As well as calibration and standardization issues. There is currently a lack of standardization in rPPG systems, which affects cross-study comparability and clinical adoption. Calibration against gold-standard methods like ECG is often required to validate the accuracy of these systems. These challenges highlight the need for further research and development to refine rPPG technologies and establish clear guidelines for their clinical use.

## 6 Conclusion

This paper presented an efficient spatio-temporal modeling-based rPPG recovery method for physiological signal measurements. The efficient spatio-temporal modeling was achieved through the 3D central difference convolution operator with a dual branch structure composed of motion and appearance, as well as a soft attention mask. Combined with Huber loss and multi-task setting, the performance was improved and the respiratory signal was also regressed. Specifically, 3D central difference convolution was adapted for temporal context modeling with enhanced representation and generalization capacity. Normalized frame difference was used as the input for motion representation, and the soft attention mask was utilized to assign a higher weight to skin areas containing physiological signals. Huber loss was deployed for robust intensity-level rPPG recovery. Through the multi-task measurement network, the pulse and respiratory signals could be measured simultaneously, which reduced the calculation cost. Extensive experiments on three public databases showed that the proposed method could resist the influence of lighting variation, movements, and skin tone to a certain extent, and outperform prior state-of-the-art methods on all three databases. The generalization ability of the model was also evaluated by cross-database experiments and video compression experiments. The effectiveness and necessity of each module in the proposed method were confirmed by ablation studies.

### 6.1 Limitations and future work

The contactless technology offers enormous promise to improve noninvasive physiological signal measurement and assessment, but it is acknowledged that considerable challenges should be overcome to accomplish this goal. A limitation of this work is that all videos in the databases are facial videos. The performance on videos with smaller facial areas or other skin regions needs further evaluation. Another limitation is that although three databases are evaluated, they are relatively small with limited environmental factors, e.g., head and facial movements, lighting, skin tone, etc. The presence of other factors may deteriorate the performance. In future work, it is planned to carry out research from two aspects: one is to analyze the effectiveness of the method under larger sample sizes and more complex factors. The other is the research on more physiological signal parameters, such as heart rate variability for broader affective computing applications. These applications can include acute stress and cognitive workload assessment (Loh et al., 2022; Debie et al., 2019).

## Data Availability

The original contributions presented in the study are included in the article/supplementary material, further inquiries can be directed to the corresponding author.

## Footnotes

1 Code available at: https://github.com/zoubochao/3DCDC-Phys
